# Formal support for informal caregivers to older persons with dementia through the course of the disease: an exploratory, cross-sectional study

**DOI:** 10.1186/s12877-016-0210-9

**Published:** 2016-01-29

**Authors:** Connie Lethin, Helena Leino-Kilpi, Brenda Roe, Maria Martin Soto, Kai Saks, Astrid Stephan, Sandra Zwakhalen, Adelaida Zabalegui, Staffan Karlsson, on behalf of the RightTimePlaceCare Consortium

**Affiliations:** Faculty of medicine, Department of Health Sciences, Lund University, Box 157, SE-221 00 Lund, Sweden; Nursing Science, University of Turku, Turku University Hospital, Turku, Finland; Evidence Based Practice Research Centre, Faculty of Health and Social Care, Edge Hill University, Ormskirk, Lancashire, UK; Alzheimer’s disease Research and Clinical Centre in Toulouse, University Hospital, Toulouse, France; Department of internal medicine, University of Tartu, Tartu, Estonia; Faculty of Health, School of Nursing Science, Witten/Herdecke University, Witten, Germany; Department of Health Services Research, Maastricht University, Maastricht, The Netherlands; Hospital Clinic of Barcelona, Barcelona, Spain

**Keywords:** Dementia, Caregivers, Health care services, Home health nursing, Nursing care, Persons, Europe

## Abstract

**Background:**

In European countries, knowledge about availability and utilization of support for informal caregivers caring for older persons (≥65 years) with dementia (PwD) is lacking. To be able to evaluate and develop the dementia support system for informal caregivers to PwD, a survey of European support systems and professionals involved is needed. The aim of this study was to explore support for informal caregivers to PwD in European countries. We investigated the availability and utilization of support in each of the participating countries, and the professional care providers involved, through the dementia disease.

**Methods:**

A mapping system was used in 2010–2011 to gather information about estimations of availability, utilization, and professional providers of support to informal caregivers caring for PwD. Data collected was representing each country as a whole.

**Results:**

There was high availability of counselling, caregiver support, and education from the diagnosis to the intermediate stage, with a decrease in the late to end of life stage. Utilization was low, although there was a small increase in the intermediate stage. Day care and respite care were highly available in the diagnosis to the intermediate stage, with a decrease in the late to end of life stage, but both types of care were utilized by few or no caregivers through any of the disease stages. Professionals specialized in dementia (Bachelor to Master’s degree) provided counselling and education, whereas caregiver support for informal caregivers and day care, respite care, and respite care at home were provided by professionals with education ranging from upper secondary schooling to a Master’s degree.

**Conclusions:**

Counselling, caregiver support, and education were highly available in European countries from diagnosis to the intermediate stage of the dementia disease, decreasing in the late/end of life stages but were rarely utilized. Countries with care systems based on national guidelines for dementia care seem to be more aware of the importance of professionals specialized in dementia care when providing support to informal caregivers. Mapping the systems of support for informal caregivers of PwD is a valuable tool for evaluating existing systems, internationally, nationally and locally for policy making.

## Background

Formal support for informal caregivers is crucial for decreasing perceived strain when caring for older persons (≥65 years) with dementia (PwD) through the trajectory of the disease. Support from health care and social services for informal caregivers may improve their wellbeing as well as ensure improved care for the older PwD [1]. Across Europe, comparable knowledge about availability and utilization of support for informal caregivers caring for older PwD is lacking. It has been suggested that to understand, interpret, and meet the needs for formal support to informal caregivers of older PwD and provide adequate dementia support/care requires professionals with specific expertise and knowledge [2, 3]. Knowledge regarding formal care providers involved in the support to informal caregivers through the dementia trajectory is sparse. To be able to develop and evaluate the dementia support system for informal caregivers of older PwD according to national policies, we need a survey of European support systems and professionals involved, which will provide a knowledge base and enable the different European countries to learn from each other.

Informal caregivers are the main providers of care and services for PwD along the dementia trajectory [4–6]. Among older PwD, informal caregivers provide approximately 75 % of the care at home including help with activities of daily living (ADLs), dealing with finances, and supervision tasks [7]. Informal caregivers may be defined as persons without formal health care education who are caring for, or helping, a person with functional disabilities, prolonged psychiatric or physical illness, or age-related problems [8]. Due to association with neurodegenerative disorders, dementia progresses along a continuum with a series of stages from diagnosis to end of life. Most cases of progressive dementia extend across more than one stage. In the early stage, there is a slight cognitive impairment, which might impact on ADLs, cognition, and social functioning. In the intermediate/moderate stage, there is increased memory loss, and dependency on help with ADLs and social needs. There is severe cognitive impairment in the late stage, when PwD are unable to look after themselves without continuous assistance with ADLs and social needs. In the end of life/final stage, it is obvious that the PwD has limited time to live [9]. The experience of informal caregivers when providing care and service to older PwD may differ from person to person. Some perceive the caring as a positive experience [10] but it can also be physically and mentally demanding and sometimes more than they can manage [10–12]. Informal caregivers caring for older PwD are at increased risk of stress, depression, strain, and other health complications [13, 14] and have higher mortality compared to informal caregivers caring for older persons without dementia [11]. Informal caregivers sometimes experience strain, and changes such as shifts in power, health, quality of life, and social networks [10, 14]. Informal caregiver strain is one reason for nursing home admission for the older PwD [15, 16]. Therefore, support from health care and social service systems is essential to empower informal caregivers to provide care at home for an older PwD through the course of the disease [17].

Significant differences exist between European countries regarding support from formal care and social services and families’ responsibilities for older PwD. In Scandinavian countries, the formal care and social support provided is based on individual needs and availability of support from the municipality or the county council [18]. In Germany, long-term care insurance only partially covers the risk of care dependency and the families are expected to contribute. The government covers all expenses in case a family is not able to contribute [19]. In Estonia, families have a legal obligation towards sick and impaired relatives, which includes provision of care and service and economic security [20]. Policies in European countries may include paid care leave (e.g. Belgium), providing respite care (e.g., Austria, Germany, and Denmark), and counselling and training services (e.g., Sweden) [21]. To be able to evaluate and develop the support for informal caregivers of older PwD, exploring the support systems in European countries is essential.

Informal caregivers have varying and individual needs of support from formal care as the dementia disease progresses [9]. The scientific evidence of the effect of different support on caregiver strain, wellbeing, and quality of life is not very strong and in some cases contradictory. In a study by Parker et al. [22], psycho-educational interventions had some positive effect on caregiver depression, health, subjective wellbeing, self-efficacy, and strain. In addition, it has been reported that support might be beneficial in decreasing caregiver strain and that multi-component interventions had varying impact on depressive symptoms, quality of life, and caregiver reaction to care recipient behaviour problems and satisfaction [23]. Another study indicated that interventions for caregivers of PwD had little effect on depression, strain, and subjective wellbeing of the informal caregiver. The interventions included psycho-educational, cognitive–behavioural, counselling/case management, and general support, and respite care [24]. Informal caregivers can have a lack of understandable knowledge [25] such as information about dementia, prognosis, and how to deal with behaviour in PwD that is perceived as difficult [26]. Professional providers of care and support (for example employed physicians, registered and auxiliary nurses) need knowledge and skills to improve the care for older PwD and the wellbeing and quality of life of the informal caregiver [27]. Hence, it is important to develop and improve the support system from formal care to ensure the wellbeing of informal caregivers. Exploring dementia care and support for informal caregivers caring for PwD through the trajectory of the disease is valuable for development of support and national policies [17].

### Aims

The aims of this study were to explore formal support for informal caregivers to older (≥65 years) PwD in terms of availability and utilization, and to examine the educational level of professional providers involved in the care and support of informal caregivers of PwD through the course of the disease across eight European countries.

## Methods

### Design

This study was an exploratory cross-sectional study conducted in eight European countries (Estonia, England, Finland, France, Germany, the Netherlands, Spain and Sweden).

### Context

The study formed a part of the European project “RightTimePlaceCare” (RTPC; the European Commission, 7th framework research project; contract number 24 21 53) with participating countries intended to represent Europe from the north, south, west, east and central [28]. The aim of the RTPC project was to improve health services for European citizens with dementia and develop best practice strategies for transition from professional home care to institutional long-term nursing care facilities. This study was carried out to find out each participating country’s health care and social service systems for informal caregivers of older PwD with regard to the country’s national regulations, guidelines, and insurances (Table 1).

**Table 1:** Care and support systems for informal caregivers to persons with dementia in eight European countries

	National regulations	National guidelines	Insurances
Estonia	Welfare services for the elderly	No national guidelines	Estonian Health Insurance Fund
Finland	The social and health care system	Under development	National Health Insurance
France	Long-term care	The Alzheimer’s disease plan “Plan AD 2008–2012”	National Health Insurance
Germany	Health care and long-term care	Three guidelines from different medical scientific societies	Long-Term Care Insurance
The Netherlands	Within the Health Insurance Act and the Social Support Act	“National Dementia Program”	Exceptional Medical Expenses Act (AWBZ): informal caregivers can apply for needs assessment
Spain	Universal Health Coverage	No national guidelines	Cohesion Fund
	Law 39/2006: promotion of personal autonomy and care of people with dependency		
Sweden	Within the Social Services Act and the Health and Medical Service Act	National guidelines for care and service for dementia	National Health Insurance
England	Within the Community Care Act and the National Health Service Act	The National Dementia Strategy for England	The National Health Service (NHS)

### The sample

Researcher collecting data for this study were RTPC partners from each participating country.

### The instrument

The mapping system was developed by Hallberg et al. [29]. It was used to describe care and service activities for PwD and their informal caregivers in the participating countries. Terminology, dementia stages and description of care and services activities for older PwD (≥65 years), informal caregivers and formal care providers involved was developed in consensus between researchers in the eight countries [29]. The mapping system horizontally described five stages of dementia: diagnosis, early stage, intermediate stage, late stage, and end of life. Vertically, different types of care and services activities were described. The mapping system included 50 different activities divided into seven categories: screening, diagnostic procedures, treatment of dementia; outpatient care facilities; care at home; institutional care; palliative care; informal caregiving and supportive actions; and civic organisations [29]. Each aspect of care and services activities, included estimations of availability, utilization, and providers of support, was related to each of the five stages of dementia. Response alternatives for estimations of availability were “[available] For all”, “For most”, “For few” and “For no one”; for estimations of utilization, they were “[utilized] By all”, “By most”, “By few”, and “By no one” [29]. The term “not applicable” (NA) was used when the activity was available, but either it was not suitable for a specific disease stage or it was not suitable at all. Providers of each care and service activity were reported in the mapping system and it was possible to state one or more care providers. In the study by Hallberg et al. [29], availability of screening, diagnostic procedures, treatment of dementia; outpatient care facilities; and palliative care was analysed. The result showed that care at home had the broadest range of activities, whilst supportive actions for informal caregiving had the smallest range. Professional care providers involved in the screening, diagnostic procedure and care at home and their educational level have been explored [30], according to the International Standard Classification of Education (ISCED) [31] (Table 2). The result showed that professionals with a Bachelor’s degree or above were involved in the screening and diagnostic procedure. Care at home was provided by professionals trained at a lower level or staff with no formal training [30]. Care and services activities available for care at home for PwD were in total 16 and are presented elsewhere [32]. In the present study, ten activities for informal caregiving and supportive actions are presented: counselling, caregiver support, education, reimbursement, no reimbursement, day care, specialized day care for dementia, residential respite care, specialized residential respite care for dementia, and respite care at home (Table 3). Professionals providing the supportive actions for informal caregiving are presented in Table 4.

**Table 2:** Categorization of professional providers of supportive actions, according to the International Standard Classification of Education (ISCED)*

ISCED LEVEL: At or above ISCED level	General health care training	Specialized health care training	Specialized training in dementia
7: Master’s or equivalent, vocational	Psychologist (psychol), provides counseling and help to people with psychological problems	GP, physician who treats patients within a district for all types of diseases	MD geriatrics (MD-ger), geriatrician or psycho-geriatrician specialized in geriatrics
		MD psychiatry^1^ (MD-psych)/Old Age Psychiatrist, specialized in psychiatry	
	Social worker (SW), provides staff management for residential care or home help service	MD neurology^1^ (MD-neuro), specialized in neurology	
		MD-internal medicine, specialized in internal medicine	
	Physiotherapist, provides rehabilitation to identify and improve, e.g., disabled movement and function		
6: Bachelor’s or equivalent, vocational	Social worker, provides staff management for residential care or home help service	Community psychiatric registered nurse, (RN-comm-psych), supports older people at home and in nursing/residential homes. Specialized in psychiatry.	RN specialized in dementia (RN dem), has an overall responsibility for dementia care in an area/municipality. Provides counseling, supervision, and assessments, and mediates contacts. Education at advanced level: Care of the elderly (1 year Master), District nurse (1 year Master), Psychiatric care (1 year Master).
		Home help officer, carries out needs assessment prior to decision about home services and care	
	Registered nurse (RN), provides care and service including help with PADLs, medical treatments, and managing the nursing care team		
		Case manager, professional (nurse or social worker) function that may include finding and outreach, comprehensive assessment and care planning, coordination of service, service provision, monitoring, and evaluation, and, in addition, meeting special needs	
	Occupational therapist (OT), provides rehabilitation to achieve optimum level of functional ability. This may include adaptation of the home and providing aids and equipment to assist with managing everyday activities.		
	Physiotherapist^2^, provides rehabilitation to identify and improve, e.g., disabled movement and function.		
	Case manager, see “Specialized health care training”		
5: Short-cycle tertiary education, vocational	Registered nurses, not Bachelor’s level		State examined nurses specialized in dementia care
			(SEN dem), not Bachelor’s level
	State examined nurse (SEN), not Bachelor’s level		
	Occupational therapist, Bachelor’s level, provides rehabilitation to achieve optimum level of functional ability. This may include adaptation of the home and providing aids and equipment to assist in managing everyday activities.		
4: Post-secondary non-tertiary, vocational	Licensed practical nurse (LPN)/auxiliary nurse (Aux-N), provides care and service including help with IADLs and PADLs, and, in addition, minor medical treatment. Health care trained at secondary school level.		
3: Upper secondary, vocational	Nurse aid/assistant nurse (Ass-N), provides care and service including help with IADLs and PADLs. Health care trained for <6 months (OECD 2005).		
	Support worker (Supp-work), home carer, psychological supporter, or home trainer paid at enhanced nursing assistant/home carer rate. Social care/nursing trained at secondary level or trained on the job.		
<3	Social worker assistant (SW-ass), performs some similar tasks as the social worker. Not trained, or trained on the job		

*International Standard Classification of Education (ISCED), 2011

^1^MD Psychiatry and MD Neurology: training in dementia is normally part of their special training

^2^Registered nurses, occupational therapists, and physiotherapists are trained at different levels in the eight participating European countries

*IADL* instrumental activities of daily living, *PADL* practical activities of daily living

**Table 3:** Availibility and utilization of supportive actions for informal caregivers in eight European countries

		Diagnosis stage		Early stage: mild cognitive impairment		Intermediate stage: moderate cognitive impairment		Late stage: severe cognitive impairment		End of life stage	
		Available for	Utilized by	Available for	Utilized by	Available for	Utilized by	Available for	Utilized by	Available for	Utilized by
Counseling: Informal and formal, professionals or agency providing support to persons with dementia (PwDs) and their family.	EE	No one	No one	No one	No one	No one	No one	No one	No one	No one	No one
	FI	All	Most	All	Most	All	Most	All	Most	All	Most
	FR	Most	Most	Most	Most	Most	Most	Few	Few	No one	No one
	DE	Most	Few	Most	Few	Most	Few	Most	Few	Most	Few
	NL	All	Most	All	Most	All	Most	All	Most	All	Most
	ES	All	Most	All	Most	All	Most	All	Most	All	Most
	SE	All	Few	All	Most	All	Most	All	Most	Few	Most
	E	Few	Few	Few	Few	Few	Few	Few	Few	Few	Few
Caregiver support: Organization where professionals provide support, such as counseling, individually and/or in groups, and provide home visits to informal caregivers.	EE	Few	Few	Few	Few	Few	Few	Few	Few	Few	Few
	FI	All	Few	All	Few	All	Most	All	Most	All	All
	FR	Few	Few	Few	Few	Few	Few	Few	Few	Few	Few
	DE	Most	Few	Most	Few	Most	Few	Most	Few	Most	Few
	NL	All	Most	All	Most	All	Most	All	Most	All	Most
	ES	Most	Most	Most	Most	Most	Most	Most	Most	Most	Most
	SE	All	Few	All	Few	All	Few	All	Few	All	Few
	E	Most	Few	Most	Few	Most	Few	Most	Few	Most	Few
Caregiver education: Training for informal caregivers providing care and service to PwDs. Education includes needs and symptoms of dementia.	EE	Most	Few	Most	Few	Most	Few	Most	Few	Most	Few
	FI	All	Few	All	Few	All	Few	All	Few	All	Few
	FR	Most	Few	Most	Few	Most	Few	Most	Few	Most	Few
	DE	All	Few	All	Few	All	Few	All	Few	All	Few
	NL	All	Most	All	Most	All	Most	All	Most	All	Most
	ES	Few	Few	Few	Few	Few	Few	Few	Few	Few	Few
	SE	Most	Few	Most	Few	Most	Few	Most	Few	Most	Few
	E	Most	Few	Most	Few	Most	Few	Most	Few	Most	Few
Informal caregivers – reimbursed: Informal caregivers employed by the public to provide care and service.	EE	No one	No one	No one	No one	No one	No one	No one	No one	No one	No one
	FI	No one	No one	No one	No one	Most	Most	Most	Most	All	All
	FR	Few	Few	Few	Few	Few	Few	Most	Most	Few	Few
	DE	Most	Most	Most	Most	Most	Most	Most	Most	Most	Most
	NL	All	Few	All	Few	All	Few	All	Few	All	Few
	ES	No one	No one	No one	No one	Most	Most	All	Most	All	Most
	SE	Few	No one	Few	Few	Few	Few	Few	Few	Few	Few
	E	All	Few	All	Few	All	Few	All	Few	All	Few
Informal caregivers – not reimbursed: Informal caregivers provide care and service voluntarily.	EE	Most	Most	Most	Most	Most	Most	Most	Most	Most	Most
	FI	Most	Most	Most	Most	Most	Most	Most	Most	Most	Most
	FR	Most	Most	Most	Most	Most	Most	Most	Most	Few	Few
	DE	Few	Few	Few	Few	Few	Few	Few	Few	Few	Few
	NL	All	Most	All	Most	All	Most	All	Most	All	Most
	ES	All	All	All	All	All	All	All	All	All	All
	SE	All	Most	All	Most	All	Most	All	Most	All	Most
	E	All	Most	All	Most	All	Most	All	Most	All	Most
Day care/day activity/day care center/day hospital: Clinic or agency providing social activities and activities to stimulate physical, mental, and intellectual functional ability, daytime.	EE	Most	Few	Most	Few	Most	Few	Few	Few	No one	No one
	FI	Few	Most	Few	Most	Few	Most	No one	No one	No one	No one
	FR	Most	Most	Most	Most	Most	Most	Few	Few	No one	No one
	DE	Few	No one	Few	Few	Few	Few	Few	Few	Few	No one
	NL	All	All	All	All	All	All	All	All	All	All
	ES	No one	No one	Most	Few	Most	Few	No one	No one	No one	No one
	SE	Most	Few	Most	Few	Most	Few	Most	Few	No one	No one
	E	Most	Few	Most	Few	Most	Few	Most	Few	Few	Few
Day care/day activity/day care center/day hospital specialized in dementia care: Clinic or agency with staff specialized in dementia care providing social activities and activities to stimulate cognitive ability, daytime. Only persons with dementia are admitted.	EE	Few	Few	Few	Few	Few	Few	Few	Few	No one	No one
	FI	Few	Most	Most	Most	Most	Most	Most	Most	No one	No one
	FR	Most	Most	Most	Most	Most	Most	Most	Most	Most	Most
	DE	Few	No one	Few	Few	Few	Few	Few	Few	Few	No one
	NL	All	All	All	All	All	All	All	All	All	All
	ES	No one	No one	Few	Few	Few	Few	Few	Few	No one	No one
	SE	Few	Few	Few	Few	Few	Few	For few	Few	No one	No one
	E	Most	Few	Most	Few	Most	Few	Most	Few	Most	Few
Respite care at home: Care at home to provide relief to informal caregivers who are caring for a family member/close friend.	EE	Few	Few	Few	Few	Few	Few	Few	Few	Few	Few
	FI	All	Few	All	Few	All	Few	All	Most	All	Most
	FR	No one	No one	No one	No one	No one	No one	No one	No one	No one	No one
	DE	All	No one	All	Few	All	Few	All	Few	All	Few
	NL	All	All	All	All	All	All	All	All	All	All
	ES	No one	No one	No one	No one	No one	No one	No one	No one	No one	No one
	SE	All	Few	All	Few	All	Few	All	Few	All	Few
	E	Most	Few	Most	Few	Most	Few	Most	Few	Most	Few

*DE* Germany, *E* England, *EE* Estonia, *ES* Spain, *FI* Finland, *FR* France, *NL* the Netherlands, *SE* Sweden

**Table 4:** Professionals providing supportive actions for informal caregivers, supportive actions and level of education*

		ISCED level 7: Master’s or equivalent, vocational	ISCED level 6: Bachelor’s or equivalent, vocational	ISCED level 5: Short-cycle tertiary education, vocational	ISCED level 4: Post-secondary non-tertiary, vocational	ISCED level 3: Upper secondary, vocational
Counseling: Informal and formal, professionals or agencies providing support to persons with dementia (PwDs) and their family.	EE					
	FI		RN, RN dem		Aux-N	
	FR	Psychologist				
	DE	Case manager^2^ psychol	Case manager^2^, SW	SEN		
	NL	Psychologist	Case manager^2^, RN dem			
	ES	MD ger, psych, neuro, GP, psychol	RN, SW			
	SE	MD ger, psych, neuro, GP, psychol				
	E	Psychologist	RN, RN dem, psych, SW, OT			Support worker
Caregiver support: Organization where professionals provide support, such as counseling, individually and/or in groups, and provide home visits to informal caregivers.	EE	Psychologist	SW			
	FI		RN, RN dem		Aux-N	
	FR		SW			
	DE		Case manager^2^			
	NL	Psychologist	Case manager^2,^, RN, SW		Ass-N	
	ES	GP, Psychologist	RN,SW			
	SE	MD ger, psych, neuro, GP, Psychol			LPN	
	E	Psychologist	RN, RN dem, psych, SW, OT			
Caregiver education: Training for informal caregivers providing care and service to PwDs. Education about needs and symptoms of dementia.	EE	MD	RN, SW			
	FI		RN, RN dem, SW		Aux-N	
	FR	MD-ger	RN, SW			
	DE		Case manager^2^			
	NL		Case manager^2^, RN, SW			
	ES	GP, Psychologist	RN, SW, OT			
	SE		RN, RN dem, SW			
	E	Psychologist	RN, RN dem, psych, SW			LPN
Informal caregivers – reimbursed: Informal caregiver employed by the public to provide care and service.	EE					
	FI		RN, RN dem, SW			
	FR	MD ger, GP	SW			
	DE					
	NL	NA^1^	NA^1^	NA^1^	NA^1^	NA^1^
	ES		SW			
	SE		SW			
	E		SW			
Day care/day activity/day care center/day hospital: Clinic or agency providing social activities and activities to stimulate physical, mental, and intellectual functional ability, daytime	EE					
	FI	GP	RN, SW, Physio-T			
	FR	MD-ger. Psychologist	RN, SW, OT, Physio-T			
	DE	SW, Physio-T	SW, OT, Physio-T	SEN	Aux-N	Ass-N
	NL	Multi-prof. team^3^				
	ES	MD-ger, psych, neuro, GP, Psychologist	RN, SW, OT, Physio-T			Ass-N
	SE		SW		Ass-N, LPN	
	E	MD-psych, Psychologist	RN, RN dem, SW, OT, Physio-T		Ass-N	
Day care/day activity/day care center/day hospital specialized in dementia care: Clinic or agency, staff specialized in dementia care providing social activities and activities to stimulate cognitive ability, daytime. Only persons with dementia are admitted.	EE		OT			
	FI		RN, SW		Aux-N	
	FR	MD-ger. Psychologist	RN, SW, OT, Physio-T			
	DE	Multi-prof. team^3^	Multi-prof. team^3^			
	NL	Multi-prof. team^3^	Multi-prof. team^3^			
	ES	MD-ger, psych, neuro, GP, psychol	RN, SW, OT, Physio-T			
	SE		SW		Ass-N, LPN	
	E	MD-ger, psych, Psychologist	RN, RN dem, SW, OT, Physio-T			Ass-N, Support worker
Respite care: For older people, but not specific to those with dementia disease: residential care around the clock for relief to informal caregivers who provide care for a family member/close friend.	EE		RN			
	FI	GP	RN		Aux-N	
	FR	GP, MD-ger	SW			
	DE		SW, OT	SEN, SEN dem		
	NL	Multi-prof. team^3^	Multi-prof. team^3^			
	ES	MD-ger, psych, neuro, GP, psychol	RN, SW, OT, Physio-T			
	SE	GP	RN		Ass-N, LPN	
	E					Support worker
Respite care, specialized in dementia care: Residential specialized dementia care around the clock for relief to informal caregivers who provide care for a family member/close friend. In a nursing home or residential home.	EE		RN			
	FI	GP	RN		Aux-N	
	FR	GP, MD-ger	SW			
	DE					
	NL	Multi-prof. team^3^	Multi-prof. team^3^			
	ES					
	SE	GP	RN		Ass-N, LPN	
	E		RN, RN dem			Support worker
Respite care at home: Care at home for relief to informal caregivers who are caring for a family member/close friend.	EE					
	FI				Aux-N	
	FR					
	DE					
	NL	Multi-prof. team^3^	Multi-prof. team^3^			
	ES					
	SE				Ass-N, LPN	
	E		RN, RN dem			Support worker

*International Standard Classification of Education (ISCED), 2011

^1^ *NA* not applicable, i.e., the support was available, but not suitable in a specific stage of the disease or not suitable at all

^2^Case managers and home health experts had some health care training at ISCED level 6 and, in addition, special training for the task

^3^The multi-professional teams commonly consist of a physician, psychologist, registered nurse, assistant nurse, occupational therapist, and physiotherapist

*DE* Germany, *E* England, *EE* Estonia, *ES* Spain, *FI* Finland, *FR* France, *NL* the Netherlands, *SE* Sweden

For abbreviations of professional titles and qualifications, see the List of abbreviations

### Data collection

Established researchers with extensive practical and research experience in dementia care from each country, seven universities and one university hospital, contributed to the data collection representing their country as a whole. The mapping system was used for collection of data on support for informal caregivers caring for PwD (≥65 years) at home, including data on availability and utilization of, and provider type for, each support service. A guide was used for data collection, which suggested consulting sources of information for public reports and descriptions of the care system, official statistics, and using personal interviews with care managers, providers, and civic administrative areas. In addition, epidemiological studies and literature reviews were used for validation of responses from professionals in each country. Data collection was completed by communication with experts (in Germany, the Netherlands, Sweden); nursing staff (England); advisory boards (Estonia, Spain, Finland, Sweden); professional care providers (Finland); and a national Alzheimer’s society (Finland) and in detail described elsewhere [28]. Data were collected between November 1^st^, 2010, and January 31^st^, 2011.

### Data analysis

Information was compiled about each country’s care and support system for informal caregivers of older PwD. Availability and utilization of support was analysed by the first author for variation, in each dementia stage, between the participating countries; thereafter, each author checked the analysis. For each country, categories of professional providers of support were compiled and documented in the template. In order to interpret education systems from a global perspective, a standardized framework - ISCED, was used to categorize and report cross-nationally to ensure comparable data [31]. The framework is categorized from level 1–7. In this study professionals were categorized from level three and above. The first author sorted the professionals into levels of education/qualifications according to ISCED’s framework. Thereafter, each author checked that the ISCED level was in agreement with their country’s educational levels (Table 2). The data sets supporting the results of this article are included within the article and its additional files.

### Ethical approval

Informed consent was collected from the participants and the study was approved by each countries Ethical Committee (with reference numbers if appropriate in brackets): Ethics Review Committee on Human Research of the University of Tartu (196/T-3), Ethical Committee of the South-West Hospital District Finland (8/2010), Comite de Protection ds Personnes Sud-Ouest and Outre-Mer Toulouse (09 202 07), Nursing Science Ethical Committee University of Witten/Herdecke, Medical Ethical Committee of the Academic Hospital Maastricht/Maastricht University (MEC 10-5-044), Ethical Committee of the Hospital Clinic Barcelona (2010/6031), Ethical Committee Lund University (20120/538), National Research Ethics Service, North West 5 Research Ethics Committee (11/NW/0003) [28].

## Results

Counselling, caregiver support, and caregiver education were the support activities with high availability from diagnosis stage to the intermediate stage, with a decrease in the late to end of life stage. Utilization was low, with a small increase in the intermediate stage (Table 3). Day care and respite care at home had the highest availability from the diagnostic to the intermediate stage, with a decrease in the late to end of life stage utilized by no or only few informal caregivers across the disease trajectory. In total, 25 types of professional support providers were identified as being involved in support activities for informal caregivers caring for an older PwD at home (Tables 4).

### Availability of support for informal caregivers

Counselling was available during all or most disease stages except in two countries (England and Estonia). Caregiver support was available for all or most stages except in two countries (Estonia and France). Caregiver education was available for all or most informal caregivers in all countries through all stages of the disease. Reimbursement was available for all or most informal caregivers in three countries in the diagnosis and early stage, increasing to five countries in the intermediate stage, and six countries in the late stage. In addition, reimbursement through the trajectory of the dementia disease was available only to few informal caregivers in two countries (Sweden and France), (Table 3). Day care was available for all or most PwD in five countries (England, Estonia, France, the Netherlands, Sweden) in the diagnosis, early and intermediate stage. In the late stage, day care was available for most patients in England and Sweden, and for few in three countries (Estonia, France, Germany). Specialized day care for dementia was available for all PwD in the Netherlands from diagnosis to the intermediate stage and for most in France and England from early stage to late stage. Respite care at home was available for all or most PwD through all stages in four countries (England, Finland, Germany, Sweden). Respite care at home was available for few across the disease trajectory in Estonia and for no one in France and Spain.

### Utilization of support for informal caregivers

Counselling was utilized by all or most informal caregivers in the intermediate to end of life stage in three countries (Spain, Finland, the Netherlands) increasing in the early to intermediate stage in four countries (Estonia, Finland, France, Sweden). Caregiver education was utilized by few in all countries except Spain and the Netherlands, where caregiver education was utilized by most informal caregivers through all stages of the dementia disease. Most informal caregivers in Germany utilized reimbursement through all stages. In England and Sweden, few got reimbursement in the early to end of life stage (Table 3). In seven out of eight countries, most informal caregivers provided care voluntarily. Day care was utilized by most informal caregivers in two countries (Finland and France) from the diagnosis to the intermediate stage and by no one or few in five countries (England, Estonia, Germany, Spain, and Sweden). Dementia specialized day care was utilized by most through all disease stages in France, while it was utilized by few or no one in the other countries. Respite care was utilized by all or most in France and the Netherlands from the diagnosis stage to the late stage of the dementia disease. In Estonia, Sweden, and England, respite care was utilized by few from diagnosis stage to the end of life stage. Respite care specialized in dementia was utilized by all informal caregivers in France through all stages of the dementia disease. In Spain, specialized respite care for dementia was utilized by no one. Respite care at home was utilized by few caregivers in England, Estonia, Spain, Germany, and Sweden, across the dementia disease trajectory.

### Professionals providing support to informal caregivers and their educational level

Counselling, caregiver support, education, and reimbursement were provided by professionals whose education ranged from upper secondary schooling to a Master’s degree in all countries (Table 4). Six out of eight countries had professionals specialized in dementia care and were able to offer this support to informal caregivers. Case managers were professionals with health care training usually at a Bachelor’s degree level. They provided support such as counselling, caregiver support, and education in Germany and the Netherlands. In Germany, state examined nurses with a short-cycle tertiary education (not a Bachelor’s degree) provided counselling to informal caregivers. In Estonia, counselling was provided voluntarily by informal caregivers and no professionals were involved. Day care and respite care (specialized and not specialized in dementia care), and respite care at home were provided by a variety of professionals with a range in education, from upper secondary schooling to a Master’s degree. In Estonia, day care (not specialized) was provided by informal caregivers and no professionals were involved. In the Netherlands, multi-professional teams were providing respite care, both with and without dementia specialization. Multi-professional teams commonly consisted of physicians, psychologists, registered nurses, assistant nurses, occupational therapists, physiotherapists, and social workers. In Germany and the Netherlands, respite care was provided by informal caregivers, with reimbursement from insurances. In Spain, respite care for dementia was unavailable. Of the four countries with national guidelines for dementia care, two had professionals with specializations in dementia care who worked in (either type of) day care.

## Discussion

This study is an innovative attempt at creating an overview and a first use of a classification system to explore support for informal caregivers to PwD through the disease course in terms of availability, utilization, and professional support providers across several countries. In this study, although availability of support for informal caregivers seems to have been high, there was low utilization of support by informal caregivers. This might be understood within the model of patient-centered access to health care. According to this model [33], there are two sides to access to the health care systems: the supply side (i.e., availability), and the demand side (i.e., utilization). Availability may be dependent on five dimensions of accessibility of services, namely: approachability; acceptability; availability and accommodation; affordability; and appropriateness. Utilization may depend on the informal caregiver’s ability to interact with the accessibility; and his or her ability to perceive, seek, reach out, pay, and engage. Access can be defined as “the opportunity to reach and obtain appropriate health care services in situations of perceived need for care” [33]. Availability and utilization of support for informal caregivers to older PwD also seems to be related to each country’s care and support systems. Four out of the eight participating countries had national guidelines for dementia care (England, France, the Netherlands, Sweden). In two more countries (Germany, Finland) these were under development at the time of data collection. Several determinants may trigger the action of accessing the health systems and utilizing care, both in the health system itself, by providers, and in the persons that need care and support.

The degree of availability of each support might depend on the characteristics of providers, organizations, and health systems. The results of this study showed that formal support for informal caregivers, from the supply side, was available in different degrees, both along the continuum of the dementia disease and across the European countries. In this study, there was high availability of counselling, caregiver support, and education from the diagnosis to the intermediate stage of the dementia disease, with decreased availability in the late to end of life stage. Access to support enables informal caregivers to enter formal care with differences in perception of needs for care, in health care seeking, in reaching and obtaining or delaying care seeking, and in type and intensity of services [33]. In a study, Morgan et al. [34] found that, at 6 months post-diagnosis, informal caregivers experienced relief, validation, and access to support and services. The diagnosis introduced the informal caregivers to support they had previously been unaware of. Even though availability of support for informal caregivers might be high, sometimes it seems that the informal caregivers do not get the information about available support. This may have an impact on the demand side, with low utilization. Low utilization can also be a result of the informal caregiver’s ability to interact with accessibility. Our results also showed that utilization of counselling, caregiver support, and education was low in the diagnosis and early stage, but increased in the intermediate stage. Reimbursement to informal caregivers was more available in the intermediate to end of life stages. There can be several explanations for these results. A previous study [35] showed that the utilization, by informal caregivers, of available respite services for frail older persons was dependent on their trust and confidence in the service. In a study investigating beliefs regarding out of home respite services showed that among informal caregivers to older PwD, non-use of respite services was strongly associated with beliefs that using the service would result in negative outcomes for the PwD [36]. It is important for each country to examine the availability of services in their care and service system in cities and provinces in order to develop and increase utilization of support to informal caregivers. A contact person/case manager [37] who can assist the informal caregiver when navigating through the care and service system may promote access and utilization on the demand side of support services. For the informal caregivers, this might build trust and confidence in formal care when caring for an older PwD through the trajectory of the disease.

Low utilization of support may be related to accessibility to the health system and, in addition, may be related to the informal caregiver’s ability to interact with the accessibility of the health system support [33]. In our study, even though availability was high, counselling, caregiver support, and education were utilized by only a few or no informal caregivers through all stages of dementia. Day care, both specialized and not specialized, and respite care at home was utilized by few or no caregivers through the disease stages. Previous studies have shown that there is a discrepancy between availability and utilization of support to informal caregivers of PwD in different stages of the disease [19, 38–41]. This may depend on access to the health system, professional providers, informal caregivers, and both users and non-users of support [33]. One study examined the difference between users and non-users of community service and results showed that the majority of informal caregivers did not participate in support groups (73 %) or use respite services (79 %). The non-users were significantly older, received less social support, and were more depressed [42]. A review [36] found that predisposing factors associated with service non-use included demographic and social structure variables, health beliefs and other beliefs, such as having high perceived duty to care or that the service was unreliable. It is important for each country to explore accessibility of its health system in order to develop and increase utilization of support to informal caregivers. An accessible health system is most important so that the informal caregiver and the older PwD may be able to receive the care and support needed. National guidelines based on current research and experience can be one way to demonstrate the benefits and risks of different interventions. Health care with national guidelines seems to ensure a specific level of care and support to all persons and mediate the health systems and organizations to strive towards established goals. To our knowledge, there are no studies exploring availability and utilization of support for informal caregivers, related to different countries’ care and support systems. However, there are studies exploring implementation of national guidelines for person-centered care, dementia strategies, and clinical guidelines for depression. Edvardsson et al. [43] implemented national guidelines for person-centered care for persons with dementia in nursing homes. Their study showed that person-centeredness of care increased from baseline to 12-month follow up. There was also a reduction in staff stress of conscience and the members of staff were able to provide the requested care and activities after the intervention. In a study of physicians in primary care settings, McKinlay et al. [44] showed that adherence to guidelines varies with different patients and the physicians’ length of clinical experience. Exploring support systems and developing national guidelines is essential to ensure quality of care and wellbeing for the informal caregiver and the PwD.

It seems that national guidelines regarding dementia care implies more professionals specialized in dementia care who provide support to informal caregivers of older PwD and meet specific needs during the trajectory of the disease. In this study, four out of eight countries had national guidelines in place for dementia care (England, France, the Netherlands, Sweden). Of these countries, two had professionals specialized in dementia care who provided counselling and caregiver support, and three had professionals with dementia specialization who provided education to informal caregivers. Specialized care professionals had education ranging from short-cycle tertiary education to Bachelor’s or Master’s degrees. Though little research seems to have been done concerning the educational level of professionals specialized in dementia care it can be assumed that higher levels of education and dementia specialization can improve the quality of care and support for informal caregivers and older PwD. One previous study showed that a higher proportion of registered nurses with a Bachelor’s degree was related to lower mortality in hospital care in nine European countries [45]. Another study [46] found that educational deficits in dementia knowledge and in knowledge about normal aging made the physicians miss the dementia diagnosis or misdiagnose the disease. They found a considerable variability in primary care providers diagnostic sensitivity (ranging from 0.26–0.60). In addition, it was found that specialists were more accurate, compared with primary care providers, when making a dementia diagnosis. Hence, it appears that to understand and properly meet the needs of older PwD and their informal caregivers, dementia care require professionals with specific expertise and knowledge [2, 3]. It has further been suggested that dementia research should focus more on the relation between the educational levels of the health professionals, and their impact on the care for older PwD, and the wellbeing and quality of life of the informal caregiver [27]. In addition, it is important to explore professional providers’ educational levels nationally to evaluate and develop the dementia support system for informal caregivers for older PwD according to national guidelines.

### Methodological limitations

This study was a first attempt to explore support for informal caregivers of older PwD in terms of availability, utilization, and providers of support (i.e., providers’ involvement and educational level) in European countries using a newly developed mapping system. The strength of this study was that we mapped data about eight European countries regarding the support for informal caregivers of older PwD in terms of availability, utilization, and providers. Our findings can serve as a knowledge base and it is hoped that they will enable the different European countries to learn from each other. The sample represents central, northern, southern, western and eastern European countries and reflects our aim to include a sample to represent Europe as a whole. However, eastern and western Europe were only represented by one country each. When developing the mapping system, the researchers from each country determined the different types of care and support for formal caregivers and agreed on the concepts and terminology. However, the collected information probably varied within organizations, regions, and countries. It was challenging to operationalize and quantify the concepts “availability” and “utilization” in a comparable way. The somewhat open and unspecific categories may have influenced the results due to different interpretations, which may reflect a range of the conditions in each country. In our study, data sources varied. The findings are not based on any research in the field and experts consulted were not systematically selected. Moreover, the term “not applicable” provided some problems. Some researchers explained that in some cases, the support was available, but not suitable for a specific stage of the disease or not suitable at all. Sometimes support was available for most, but was utilized by few without any relation between availability and use. All participants were instructed how to understand the meaning of the concepts “availability” and “utilization” as well as how to assess the care in relation to the stages of the disease for validation of the data previously collected [29]. To establish reliability, each country’s researchers were given the same instructions for data collection. The response alternatives for “availability” were “For all”, “For most”, “For few” and “For no one”, and for utilization, “By all”, “By most”, “By few”, and “By no one”. Responses were estimations, without any statistic basis. Each country’s researchers reported their country’s health systems and policies for dementia care and services, assessed and interpreted from their cultural and societal perspective, which may have affected the validity of the results. The weakness of this study may therefore be the data collection method, which allowed room for different cultural interpretations. Since data used in this study was collected between November 1^st^, 2010, and January 31^st^, 2011, it is possible that the result may have been different if data were collected today. Support systems for informal caregivers to older PwD might have been developed in participating countries to improve support for informal caregivers. In addition, professionals with dementia specialization might have increased to improve both support to informal caregivers and quality of care for PwD. Future studies using the mapping system on a national level, cities or provinces, will make it possible to improve inter-rater agreement with fewer data-collectors. National and more local mapping will improve the representative of samples (e.g. organisations and services providers) and make it possible to collect primary and more precise and statistical data regarding availability and utilisation. In future studies it will be important to consider differences between urban and rural areas and populations with different social-economic statues and ethnical backgrounds.

## Conclusion

Counselling, caregiver support, and education were highly available from diagnosis to the intermediate stage of the dementia disease, decreasing in the late and end of life stages. These support activities were, however, utilized by few or no caregivers to PwD across the disease trajectory. Estimations of availability and utilization of support for informal caregivers appears to be closely related to each country’s care and support systems. Countries with national guidelines for dementia care seemed to be more aware of the importance of having professionals specialized in dementia care involved in providing support to PwD and their informal caregivers. The mapping system used in this study to identify the support for informal caregivers of older PwD is valuable for evaluating this support system, both nationally and locally. Applying the mapping system on a local level (cities and provinces) will make the care and support system through the course of the dementia disease visible and ensure that adequate care and support are offered to informal caregivers to PwD. In addition, the mapping system may also be useful for informing the development of policy for care and services for PwD and their informal caregivers.

## Abbreviations

ADL: Activities of daily living
Ass-N: Nurse aid/assistant nurse
Aux-N: Auxiliary nurse
DE: Germany
E: England
EE: Estonia
ES: Spain
FI: Finland
FR: France
ger: Geriatrics
IADL: Instrumental activities of daily living
ISCED: The International Standard Classification of Education
LPN: Licensed practical nurse
MD: Medical doctor
MD-ger: Medical doctor specialized in geriatrics
MD-neuro: Medical doctor specialized in neurology
MD-psych: Medical doctor specialized in psychiatry
NL: The Netherlands
OT: Occupational therapist
PADL: Practical activities of daily living
physio-T: Physiotherapist
psych: Psychiatry
psychol: Psychology
PwD: Person with dementia
RN: Registered nurse
RN-comm-psych: Community psychiatric registered nurse
RN dem: Registered nurse specialized in dementia
RTPC: RightTimePlaceCare
SE: Sweden
SEN: State examined nurse
SEN dem: State examined nurse specialized in dementia
supp worker: Support worker
SW-ass: Social worker assistant
SW: Social worker
